# Pre-attentive fundamental frequency processing in Mandarin-speaking children with cochlear implants as revealed by the peak latency of positive mismatch response

**DOI:** 10.3389/fnins.2022.1045939

**Published:** 2022-12-08

**Authors:** Wanting Huang, Lena L. N. Wong, Fei Chen

**Affiliations:** ^1^Department of Electrical and Electronic Engineering, Southern University of Science and Technology, Shenzhen, China; ^2^Unit of Human Communication, Development, and Information Sciences, Faculty of Education, The University of Hong Kong, Hong Kong, Hong Kong SAR, China

**Keywords:** fundamental frequency, cochlear implant, mismatch response, children, peak latency

## Abstract

**Introduction:**

Fundamental frequency (F0) serves as the primary acoustic cue for Mandarin tone perception. Recent behavioral studies suggest that F0 information may be differently processed between Mandarin-speaking normal-hearing (NH) children and children with cochlear implants (CIs), which may partially explain the unsatisfactory outcome of lexical tone recognition using CIs with tonal language-oriented speech processing strategies. The aim of the current study was to provide neural evidence of F0 processing in Mandarin-speaking kindergarten-aged children with CIs compared with NH children.

**Methods:**

Positive mismatch responses (p-MMRs) to the change of the two acoustic dimensions of F0 (F0 contour and F0 level) in Mandarin-speaking kindergarten-aged children with CIs (*n* = 19) and their age-matched NH peers (*n* = 21).

**Results:**

The two groups of children did not show any significant difference on the mean amplitude of p-MMR to either F0 contour or F0 level change. While the CI group exhibited a significantly shorter peak latency of p-MMR to F0 contour change than to F0 level change, an opposite pattern was observed in the NH group.

**Discussion:**

This study revealed a higher sensitivity to F0 contour change than to F0 level change in children with CIs, which was different from that in NH children. The neural evidence of discrepant F0 processing between children with CIs and NH children in this study was consistent with the previously reported behavioral findings and may serve as a reference for the development and improvement of tonal language-oriented speech processing strategies.

## Introduction

According to the Seventh National Census in 2020, more than 800,000 children under the age of seven have been diagnosed with severe to profound hearing impairment in mainland China, with an annual growth of prelingually deaf children ranges from 16,000 to 48,000 (Nin, 2021). In recent decades, cochlear implantation has been a common treatment for Mandarin-speaking children with severe to profound hearing impairment. Benefit from the National Cochlear Rescue Project, about 2,000 hearing-impaired preschool children in mainland China had received implantation in 2004, with an increasing rate of 30–50% per year thereafter (Du et al., 2005).

The most prominent difference between tonal and non-tonal languages lies in the use of rapid pitch variations, i.e., lexical tones, to alter word meanings. In tonal languages, fundamental frequency (F0) is the primary acoustic cue for lexical tone recognition (Lin, 1988). The conventional speech processing strategies (e.g., continuous interleaved sampling, advanced combination encoder) in CI systems typically conveys the temporal envelope of sound stimuli to CI users. Given the limited access to F0 information, speech perception, especially in noise conditions, remains unsatisfactory in Mandarin-speaking CI users (e.g., Wei et al., 2007; Chen et al., 2014). In recent years, tonal language-oriented speech processing strategies have been developed to provide more F0 information by delivering more spectral information (e.g., the HiResolution 120 strategy; Wilson, 2006) or fine structure cues (e.g., the Fine Structure Processing strategy; Hochmair et al., 2006). Unfortunately, these tonal language-oriented speech processing strategies failed to demonstrate significant improvement of Mandarin tone recognition in CI users when compared to that with conventional speech processing strategies (e.g., Han et al., 2009; Qi et al., 2017). For example, Han et al. (2009) investigated the effectiveness of the HiRes 120 strategy on Mandarin tone recognition in a group of 12 prelingually deaf children (aged 3.5–16.5 years). Unfortunately, despite 6 months’ conversion from the conventional strategy (i.e., the HiRes strategy), no statistically significant improvement was seen from Mandarin tone recognition scores in these participants (Han et al., 2009). It is well recognized that there is a critical period for language acquisition and learning during preschool age (McCarthy, 1943; Berk, 2003; Conti-Ramsden and Durkin, 2012). Therefore, better understanding of F0 processing in Mandarin-speaking preschool children with cochlear implants (CIs) may provide valuable references for the improvement of speech processing strategies targeted at tonal language-speaking CI users.

From the perspective of acoustic perception, F0 comprises two elements: F0 contour and F0 level (Gandour, 1983). F0 contour describes the shape and direction of the continuous pitch change and plays a major role in distinguishing word meanings (Clark and Yallop, 1990; Yip, 2002). In contrast, F0 level reflects step changes of sound pitch from the onset, which is responsible for discriminating different talkers (Yip, 2002). Unfortunately, the processing of F0 contour and F0 level has not been well documented until recent years. To date, the few studies reporting F0 processing in Mandarin-speaking children with CIs have focused mainly on the effect of the F0 contour on Mandarin tone perception (e.g., Peng et al., 2017; Zhang et al., 2018; Huang et al., 2020). For example, by systematically manipulated the F0 contours and durations of a Chinese disyllabic word (*yanjing*) in a lexical tone identification recognition task, Peng et al. (2017) investigated acoustic cue processing in 28 Mandarin-speaking listeners with CIs (aged 6.6–21.4 years) and 11 age-matched (aged 4.6–15.6 years) peers with normal hearing. As indicated by the regression coefficient in the simple logistic regression models, which was used to approximate children’s reliance on F0 contours/durations in tone identification, children with CIs relied more on duration cues than on F0 contours, exhibiting an opposite pattern to that in age-matched normal-hearing (NH) peers. In contrast, at sentence level, Huang et al. (2020) reported that when the other acoustic cues (e.g., temporal envelope) were neutralized, the effects of F0 contours on sentence recognition in Mandarin-speaking children with CIs (aged 3.42–6 years) was significantly more salient than those in NH children in both quiet and noise. Despite the seemingly contrasting effects of F0 contours on Mandarin tone recognition reported at word and sentence level, findings in Peng et al. (2017) and Huang et al. (2020) consistently suggest that processing of F0, at least F0 contours, in children with CIs may be different from that in NH children. This conclusion is further supported by a recent study, which found that while Mandarin-speaking preschool children with CIs were more sensitive to F0 contour change than to F0 level change (i.e., smaller just-noticeable differences of F0 contour change compared to that of F0 level change), an opposite pattern of sensitivity to F0 contour and F0 level change was observed in the age-matched NH group (Huang et al., 2022).

According to the search of the existing literature from the electronic databases (i.e., Wiley Online Library, Scopus, Pubmed) from 1980 to September 2022, F0 processing in Mandarin-speaking children with CIs has mostly been investigated at the attentive stage (i.e., requiring attention when performing tasks), and discrepant findings were reported across studies (e.g., Peng et al., 2017; Huang et al., 2022). In recent decades, the event-related potential (ERP) technique has been applied to auditory research in pediatric CI users, including music and lexical tone perception (Kileny et al., 1997; Singh et al., 2004; Henkin et al., 2008; Torppa et al., 2012; Liang et al., 2014; Sharma et al., 2015; Fu et al., 2016). Numerous studies have demonstrated that MMRs, which reflect automatic stimuli discrimination at the pre-attentive stage (i.e., no attention or behavioral responses are required for completing tasks) (Segalowitz and Barnes, 1993; Luck, 2014) are reliable biomarkers predicting behavioral performance (Tiitinen et al., 1994; Benasich et al., 2006; Perez et al., 2017; Uhler et al., 2021). Accordingly, in this study, MMRs are employed as the electrophysiological indicators of F0 processing in Mandarin-speaking children with CIs at the pre-attentive stage. It is believed that findings in the current study would not only help to reveal F0 processing in Mandarin-speaking children with CIs at an earlier processing stage (i.e., pre-attentive vs. attentive stage), but also draw a more comprehensive picture on this research question together with the existing behavioral findings.

Mismatch responses are usually elicited with the oddball paradigm, in which stimulus sequence is composed of frequently presented standard stimuli and infrequently presented deviant stimuli. MMRs are obtained by subtracting the ERPs elicited by standard stimuli from those elicited by deviant stimuli (Näätänen et al., 2007; Nan et al., 2016). According to existing literature, the polarities of MMRs may exhibit as either negative [i.e., mismatch negativity (MMN)] or positive [i.e., positive mismatch response (p-MMR)]. Since its discovery in late 1970s (Näätänen et al., 1978), MMN has been extensively used as an electrophysiological index for automatic detection of auditory stimulus change in adults, peaking around 100–250 ms after stimulus onset (e.g., Pang et al., 1998; Light et al., 2007; Näätänen et al., 2007; Wang et al., 2013; Nan et al., 2016; Schwade et al., 2017). In recent years, MMN to lexical tone (Fu et al., 2016) or pitch (Kileny et al., 1997; Torppa et al., 2012; Liang et al., 2014) change has also been reported in children with CIs, in which MMN was observed in a later time window than that in NH adults (i.e., as long as 400 ms following stimulus onset). Amplitude and peak latency of MMN can be used to indicate discrimination ability of auditory stimuli (Näätänen et al., 2007), which means that for given standard and deviant stimuli, better discrimination ability in individuals is associated with larger amplitude and shorter peak latency (Näätänen, 2001, 2008; Cheour et al., 2002; Fu et al., 2016). For instance, to investigate the effect of hearing experience (i.e., NH vs. CIs) on change detection of lexical tone (rising tone vs. falling tone) change, Fu et al. (2016) compared amplitude and peak latency of the evoked MMN responses between 20 Mandarin-speaking preschool children with normal hearing and 40 children with either binaural hearing aids or unilateral implantation. Results showed that children with hearing impairment exhibited longer peak latency and smaller amplitude of MMN compared to those in NH children, suggesting that lexical tone perception at cortical level was significantly compromised in children with hearing impairment.

When applying the oddball paradigm, some studies reported that p-MMR, rather than MMN, was observed in preschool and primary school children on pitch change detection, with peak latency of p-MMR varying from 180 to 450 ms after stimulus onset (Shafer et al., 2000; Maurer et al., 2003; Ahmmed et al., 2008; Lee et al., 2012). Although both MMN and p-MMR reflect automatic change detection at the pre-attentive stage, there has long been a debate on which type of MMR is to expect when measuring children participants. One hypothesis on this issue proposes that there is a developmental transition from p-MMR to MMN, which reflects neural maturation of human brain (Trainor et al., 2001; He et al., 2007, 2009a,b; Cheng et al., 2013; Liu et al., 2014). For example, Liu et al. (2014) compared MMRs to lexical tone change (rising tone vs. dipping tone) in NH preschoolers, school-age children, and adults. Results showed that while MMN was observed in adults, p-MMR was discovered in preschoolers, and MMR to lexical tone change in school-age children was in-between (Liu et al., 2014). Similar findings were reported in a recent study. Engström et al. (2021) conducted a follow-up study on the pitch-evoked mismatch responses in a group of Swedish-speaking children with bilateral CIs or a CI combined with HA. Harmonic tones composed of 500, 1,000, and 1,500 Hz sinusoidal partials were employed as standard stimuli, and the pitch of the deviant stimuli was 10% higher or lower than that of the standard. MMN was observed when these children were of school age (mean = 9.1 years, ranged from 8.9 to 9.5 years), which was hardly seen when they were preschool children (mean = 6.4 years, ranged from 5.8 to 6.9 years). Another hypothesis on the polarity of MMRs in oddball paradigm argues that morphology of MMRs is related to the characteristics of auditory stimuli (Morr et al., 2002, Lee et al., 2012; Cheng et al., 2013, 2015; Partanen et al., 2013; Tong et al., 2014; Uhlén et al., 2017). For example, Tong et al. (2014) investigated MMRs to pitch and duration change in a group of Cantonese-speaking NH children aged between seven and eight. Researchers found that pitch change elicited significant p-MMR response, while detection of duration change was reflected by MMN response (Tong et al., 2014). Such MMRs to pitch and duration change was also reported in English-speaking NH children aged between 5 and 7 years old (Uhlén et al., 2017).

The aim of the current study was to investigate F0 contour and F0 level processing at the pre-attentive stage in Mandarin-speaking preschool children with CIs compared to their age-matched NH peers. The ability to detect F0 contour and F0 level change in these children was reflected by the amplitude and peak latency of MMRs. According to previous studies, p-MMRs were observed when assessing pitch change detection in NH preschoolers speaking either tonal or tonal language (Tong et al., 2014; Uhlén et al., 2017). Therefore, in this study, it was expected that p-MMRs would be observed in Mandarin-speaking preschool children when F0 contour/F0 level change was detected. Amplitude and peak latency of p-MMRs to F0 contour and F0 level change were recorded and compared between the NH group and the CI group. Existing studies have demonstrated that larger amplitude and/or shorter peak latency of MMRs to speech/non-speech auditory change were positively correlated with better performance in behavioral tasks among CI users (Singh et al., 2004; Dinces et al., 2009; Ibraheem et al., 2020). Thus, based on the well-known compromised F0 information provided by CI systems, as well as the less reliance on F0 contour for lexical tone recognition in Mandarin-speaking children with CIs compared to NH children (Peng et al., 2017) at the attentive stage, it was expected that p-MMRs to F0 contour and F0 level change in children with CIs had smaller amplitude and/or longer peak latency than those in NH children.

## Materials and methods

### Participants

Children in the CI group consisted of 19 children (10 boys and 9 girls, mean age = 4.25 years old, ranged from 3.42 to 5.75 years old). All were recruited from the China Rehabilitation Research Center for Hearing and Speech Impairment. Demographic information about children with CIs is shown in Table 1. All children exhibited congenital bilateral severe to profound sensorineural hearing loss and received unilateral implantation. Except for one child (No. 1), CIs used by children in this group were produced by one of the three major manufacturers of CIs: Advanced Bionics (AB), Cochlear, and MED-EL. Either Phonak or Widex hearing aids had been fitted and worn unilaterally (*N* = 4) or bilaterally (*N* = 6) before implantation. After implantation, all children wore hearing aids on their unimplanted ears as bimodal fittings. A group of age-matched children (11 boys and 10 girls, mean age = 4.41 years old, aged from 3.33 to 5.5 years old), whose audiometric thresholds were equal to or below 20 dB HL at octave frequency between 250 and 4,000 Hz, were recruited as normal controls. Children were trained to raise their right hands upon hearing the “beep,” the procedure of which was the same to that in the pure-tone test. All children were native speakers of Mandarin Chinese and scored within normal range on the Hiskey–Nebraska Test of Learning Aptitude for children above 3 years old (Hiskey, 1956). The research protocol was approved by the Human Research Ethics Committee of the University of Hong Kong. All children voluntarily participated in this study, with written informed consent obtained from parents.

**TABLE 1:** Demographics of children with CIs.

No.	Gender	AAT (years)	HAT (years)	AAI (years)	Acoustic device			DCI (years)	DAVT (years)
							
					Left	Right	Speech processing strategies		
1	F	4.83	Null	2.58	Phonak	Nurotron	C-tone	2.25	1.91
2	M	4.33	0.33	1.42	Phonak	AB	HiRes 120	2.91	2.91
3	M	3.58	Null	0.67	Phonak	AB	HiRes 120	2.91	2.08
4	F	5.75	1.58	3.58	Phonak	Med-EL	FS4	2.17	2.17
5	M	3.5	Null	1.67	Phonak	Med-EL	FS4	1.83	1.25
6	M	4.33	0.75	3.25	Phonak	Cochlear	ACE	1.08	1.08
7	M	3.92	Null	1.33	Phonak	Med-EL	FS4	2.59	2.5
8	M	5.33	Null	3.17	Phonak	Med-EL	FS4	2.16	2.17
9	M	3.5	Null	1.58	Phonak	Cochlear	ACE	1.92	1.92
10	F	3.42	0.67	2.08	Med-EL	Phonak	FS4	1.33	1.33
11	M	4.17	Null	3	Med-EL	Phonak	FS4	1.17	1.17
12	F	4.17	0.67	1.58	Cochlear	Widex	ACE	2.59	1.58
13	M	3.83	0.67	1.92	Cochlear	Phonak	ACE	1.92	1.92
14	F	4.08	1	3.33	Med-EL	Phonak	FS4	0.75	0.75
15	F	5.08	0.42	3.33	Phonak	Cochlear	ACE	1.75	1.75
16	F	4	0.33	2.75	Phonak	Cochlear	ACE	1.25	1.25
17	F	4.83	0.83	2.92	Cochlear	Phonak	ACE	1.92	1.75
18	F	4.08	Null	2.58	Med-EL	Phonak	FS4	1.5	1.33
19	M	4	Null	2.08	Phonak	Cochlear	ACE	1.92	1.67

AAT, age at test; HAT, hearing aid trial before implantation; AAI, age at implantation; DCI, duration of CI use; DAVT, duration of auditory-verbal training; AB, Advanced Bionics; FS4, Fine Structure 4; ACE, advanced combination encoder; HiRes 120, HiResolution 120.

### Stimuli

An isolated vowel/a/with tone 1 was first recorded from an adult woman, who was a native Mandarin speaker. The F0 contour of/a1/was then replaced with a series of linear F0 contours using the Praat software (Boersma and Weenink, 2008), with other acoustic features remaining the same. Eight F0 contours were generated in the block measuring neural responses to F0 contour change, including seven flat contours (i.e., 100, 133, 167, 200, 233, 267, and 300 Hz) and one rising contour (i.e., onset F0 at 100 Hz and offset F0 at 300 Hz). In contrast, only two flat contours were used in the block measuring neural responses to F0 level change, namely 100 and 300 Hz. The selection of the frequency range was based on Huang et al. (2022), in which frequency change within 100–300 Hz was discriminable in Mandarin-speaking preschool children with CIs. According to the Syllabus of the Chinese Proficiency Test, the duration of a naturally uttered monosyllabic word lasts about 200–300 ms (Liu, 1989). To ensure the ecological validity and audibility of stimuli, the durations of each stimulus in both F0 contour and F0 level condition were set at 300 ms.

### Procedure

**FIGURE 1:**
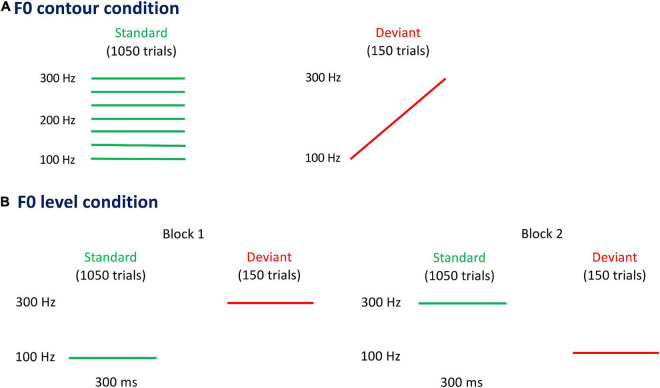
Pitch contours carried by vowel/a/in fundamental frequency (F0) contour condition **(A)** and F0 level condition **(B)**. In the F0 contour condition, seven flat F0 contours (left panel) carried by vowel/a/are used as standard stimuli, each of which presents with 12.5% probability (i.e., 150 trials). The rising F0 contour (right panel) carried by vowel/a/is used as deviant stimulus, being presented with 12.5% probability (i.e., 150 trials). There are two blocks in the F0 level condition, in which the two flat F0 contours carried by vowel/a/are used as standard stimulus in one block and as deviant stimulus in the other block. The probability of standard and deviant stimuli in each block is 87.5% (i.e., 1,050 trials) and 12.5% (i.e., 150 trials), respectively.

The p-MMR was elicited using the passive oddball paradigm (Näätänen et al., 2007). As is shown in Figure 1, the F0 contour condition and the F0 level condition composed of one and two experimental blocks, respectively. The stimulus sequence in each experimental block contained 1,200 trials, including 1,050 trials (i.e., 87.5% probability) of standard stimuli and 150 trials (i.e., 12.5% probability) of deviant stimuli. The deviant stimuli were pseudo-randomly inserted among standard stimuli, with every two adjacent deviant stimuli being separated by at least two standard stimuli. The inter-stimulus interval was set at a random length between 600 and 800 ms. In the F0 contour condition, to avoid the possibility that processing of F0 contour was contaminated by the F0 level and other acoustic features, seven flat F0 contours (i.e., 100, 133, 167, 200, 233, 267, and 300 Hz) carried by vowel/a/were used as standard stimuli, each of which was presented at an equal probability (12.5%). Vowel/a/with a rising contour, linearly rising from 100 Hz to 300 Hz, was chosen as the deviant stimulus. In the F0 level condition, there were two flat F0 contours carried by vowel/a/, i.e., 100 and 300 Hz. The 100 and 300-Hz flat F0 contours were, respectively, employed as standard stimuli in the two blocks.

Children were seated in a soft and firm sofa in an electrically shielded and soundproof room. During the test, children in the CI group wore their CIs only, with the hearing aids on the contralateral ear turned off and removed from ears. Sound stimuli were presented at a listening level of 65 dB sound pressure level (SPL) *via* a loudspeaker located in front of children at a 0° azimuth and a distance of one meter. Along with the presentation of sound stimuli, silent cartoons of interest were performed on a computer screen, which was right above the loudspeaker. Children were asked to ignore the sound stimuli and minimize eye and body movements while watching cartoons. The three blocks were presented in a random order for each child. Each block lasted 20 min, followed by a 5-min break.

### Electroencephalographic recording

**FIGURE 2:**
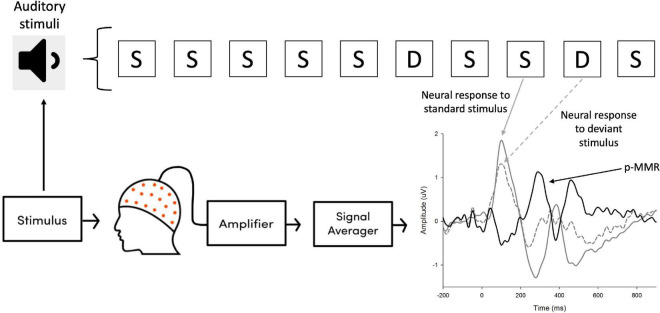
The signal recording paradigm in this study. Neural responses evoked by the auditory stimuli were amplified by the amplifier, which was then processed and averaged across trials. The positive mismatch response (p-MMR) was obtained by subtracting the event-related potentials (ERPs) elicited by standard stimuli (S) from those elicited by deviant stimuli (D).

The electroencephalographics (EEGs) were recorded using an electrocap with 64 channels, which were placed according to the international 10–20 system. The signal recording paradigm was shown in Figure 2. Visualization of real-time EEG recording was realized by the Scan 4.5 package (Neuroscan, Walnut Creek, CA, USA) and a SynAmps amplifier. The band-pass filter of EEG recording was set at 0.05–200 Hz with a 1,000-Hz sampling rate. The electrooculograms (EOGs) activity were recorded above and below the left eye (i.e., vertical EOG) and next to the outer canthi of left and right eyes (i.e., horizontal EOG). The tip of nose was chosen as the reference point in this study, with the reference electrode attached on it. The impedance of all electrodes was kept below 10 kΩ.

### Data analysis

All children completed three blocks of recording. The off-line analysis of EEG data was conducted with the EEGLAB 14.01, which was an open source for electrophysiological data processing running on the Matlab 9.5 (R2018b; The Mathworks, MA, USA). Upon EEG data import, trials with saccades were first removed by visual inspection. The remaining EEG data was then digitally filtered, with the high-pass and low-pass filter being 0.1 and 30 Hz, respectively. Next, the continuous EEG data was further divided into 1,100-ms epochs, including 200-ms pre-stimulus onset and 900-ms post-stimulus onset. Following baseline correction, which used the 200-ms pre-stimulus brain waves as standards, EEG data in each child was decomposed into 40 independent components using the independent component analysis (ICA) algorithm. Independent components that represented vertical and horizontal eye movements and CI electrical artifacts, as indicated by the 2-D voltage map and the activity spectrum, were rejected by visual inspection. Preprocessing of EEG data is completed by recombining the remaining independent components. Approximately, 70% of trials remained after artifact rejection.

The grand-averaged waveforms elicited by standard and deviant stimuli were obtained in each child and each electrode. In the F0 contour condition, neural responses to standard stimuli were obtained by averaging the waveforms elicited by seven flat F0 contours. In the F0 level condition, neural responses to standard and deviant stimuli were calculated as the averaged waveforms that, respectively, obtained from block 1 and 2. The p-MMRs were calculated by subtracting averaged waveforms elicited by the standard stimuli from that elicited by the deviant stimuli.

**FIGURE 3:**
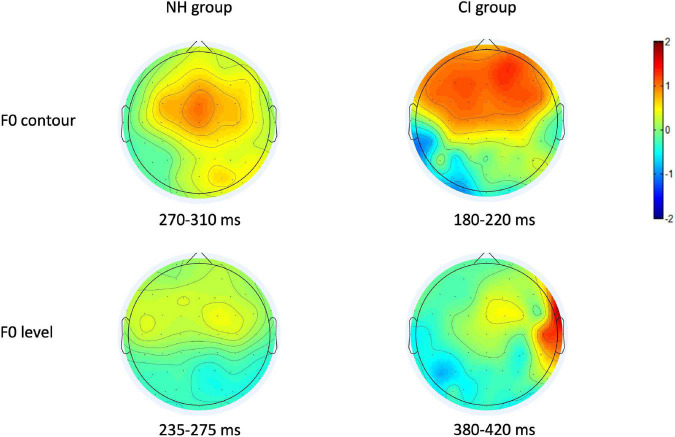
The positive mismatch response (p-MMR) amplitude scalp distributions in the selected time windows for fundamental frequency (F0) contour and F0 level conditions in the normal-hearing (NH) group and the CI group.

Consistent with previous studies (Näätänen et al., 2007; Debener et al., 2008), visual inspection on the topography of p-MMR showed that fronto-central region of brain cortex exhibited the largest amplitudes, except for the CI group in the F0 level condition (see Figure 3). Therefore, nine electrodes that were located in the fronto-central region, i.e., F3, Fz, F4, FC3, FCz, FC4, C3, Cz, and C4 were selected as electrodes of interest, and the amplitudes and peak latencies of p-MMRs among these nine electrodes were, respectively, averaged and analyzed.

## Results

**FIGURE 4:**
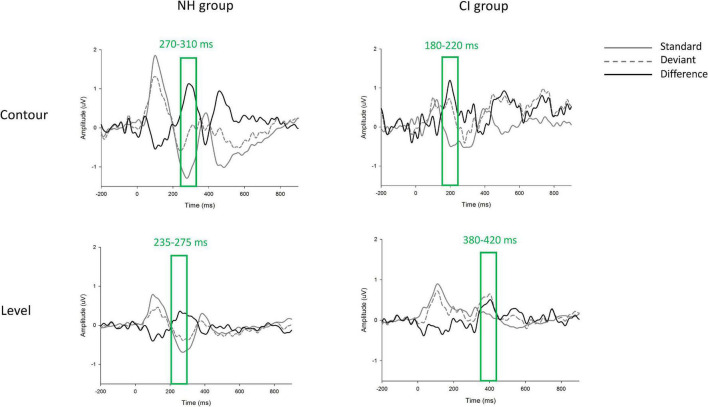
The grand-averaged waveforms elicited by standard (gray solid line) and deviant (gray dashed line) stimuli at electrode FCz. The difference wave, i.e., positive mismatch responses (p-MMRs), were shown in black solid line. Time windows in each condition and each group were marked with rectangles.

The group-level grand-averaged waveforms elicited by standard and deviant stimuli, as well as the difference waves between standard and deviant waveforms (i.e., the p-MMRs) at electrode FCz are shown in Figure 4. Based on the waveforms of p-MMRs averaged across the nine selected electrodes, time windows in F0 contour and F0 level conditions in the NH group were 270–310 ms and 235–275 ms post stimulus, respectively, and those in the CI group were 180–220 ms and 380–420 ms post stimulus, respectively. In both groups, the peak latency of p-MMR was measured as the time (from the onset of stimuli) required for p-MMR amplitude to reach its maximum within the selected time window. The p-MMR amplitudes elicited by F0 level and F0 contour change were calculated as the mean amplitudes across the selected 40-ms time window, which centered on the peak of the waveform within the time window.

### Mean amplitudes

A two-way analysis, with group (NH/CI) as the between-subject factor, and F0 dimension (F0 contour/F0 level) as the within-subject factor, was conducted to find out the effects of hearing experience (i.e., NH vs. CIs) and F0 dimension (i.e., F0 contour vs. F0 level) on p-MMR amplitudes. Statistical analysis did not reveal any significant main effect of group [*F*_(1, 38)_ = 0.62, *p* > 0.05], F0 dimension [*F*_(1, 38)_ = 0.94, *p* > 0.05], or interaction between group and F0 dimension [*F*_(1, 38)_ = 2.14, *p* > 0.5].

### Peak latencies

**FIGURE 5:**
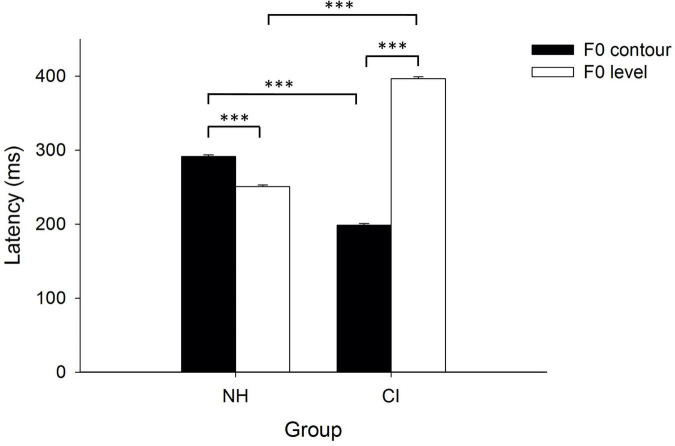
Peak latencies of positive mismatch responses (p-MMRs) as a function of fundamental frequency (F0) contour and F0 level in both groups. The error bar denotes one standard error of the mean. NH: children with normal hearing; CI: children with CIs. ***p < 0.001.

A two-way analysis, with group (NH/CI) as the between-subject factor, and F0 dimension (F0 contour/F0 level) as the within-subject factor, was conducted to find out the effects of hearing experience (i.e., NH vs. CIs) and F0 dimension (i.e., F0 contour vs. F0 level) on peak latencies of p-MMRs. Results indicated significant main effects of group [*F*_(1, 38)_ = 129.03, *p* < 0.001, partial η^2^ = 0.77] and F0 dimension [*F*_(1, 38)_ = 1,082.44, *p* < 0.001, partial η^2^ = 0.97], and interaction between group and F0 dimension [*F*_(1, 38)_ = 2,494.12, *p* < 0.001, partial η^2^ = 0.99]. Simple effect test on the interaction between group and condition found that peak latencies of p-MMRs significantly differed between children with CIs and NH children in both F0 contour [*F*_(1, 38)_ = 948.01, *p* < 0.001, partial η^2^ = 0.96] and F0 level [*F*_(1, 38)_ = 2494.12, *p* < 0.001, partial η^2^ = 0.98] conditions. Moreover, in the NH group, peak latency of p-MMR in F0 contour condition was significantly longer than that in F0 level condition [*F*_(1, 38)_ = 152.83, *p* < 0.001, partial η^2^ = 0.80], while in the CI group, peak latency of p-MMR in F0 contour condition was significantly shorter than that in F0 level condition, [*F*_(1, 38)_ = 3,267.97, *p* < 0.001, partial η^2^ = 0.99], which was even shorter than the peak latency of p-MMR in F0 level condition in NH children (see Figure 5).

## Discussion

F0 contour and F0 level are the two important acoustic features that make up F0, which is the primary acoustic cue for Mandarin tone recognition (Lin, 1988). To the best of our knowledge, this is the first study investigating F0 processing in Mandarin-speaking preschool children with CIs at the pre-attentive stage. Results in the current study showed that amplitudes of p-MMRs did not show significant difference between children with CIs and NH children; rather, peak latencies of p-MMRs significantly differed between both groups and conditions. In NH children, the peak latency of p-MMR to F0 contour change was significantly longer than that to F0 level change, which was opposite to the situation in children with CIs. Moreover, the peak latency of p-MMR to F0 contour change in children with CIs was the shortest across the two conditions in two groups. The opposite pattern of change detection of F0 contour and F0 level change between children with CIs and NH children suggested different F0 processing in these two groups of children. In addition, the outstanding sensitivity to F0 change at the pre-attentive stage in children with CIs, as reflected by the relatively short peak latency of p-MMR in the F0 contour condition, seemed to be inconsistent with previous behavioral findings in Peng et al. (2017), which indicated that there might be a dissociation on F0 contour perception between pre-attentive and attentive stage.

In a previous study measuring just-noticeable differences (JNDs) of contoured pitch and level pitch change in Mandarin-speaking NH adults, researchers found that JND of contoured pitch change was significantly larger than that of level pitch change, suggesting that adults with normal hearing were more sensitive to level pitch change (Huang et al., 2015). Similar findings were also reported in electrophysiological studies. For example, Wang et al. (2013) compared peak latencies of MMNs to pitch level and pitch contour change in twelve Mandarin-speaking NH adults and found that detection of pitch level change was significantly faster than that to pitch contour change. In this study, higher sensitivity to F0 level change than to F0 contour change in NH children, as reflected by the significantly shorter peak latency of p-MMR to F0 level change, was consistent with findings in Wang et al. (2013) and Huang et al. (2015). Except for the difference on peak latencies of MMNs to F0 contour and F0 level change, Wang et al. (2013) also reported a hemispheric lateralization for change detection of pitch level and pitch contour change in NH adults. While right hemisphere was responsible for pitch level change detection, pitch contour processing was more lateralized to the left hemisphere (Wang et al., 2013). In the current study, the lateralization effect of F0 contour and F0 level processing was also investigated in NH children; however, paired *t*-tests of peak latencies of p-MMRs to F0 level/F0 contour change between left (electrodes F3, FC3, and C3) and right (electrodes F4, FC4, and C4) hemisphere did not yield any significant difference (all *p*s > 0.05), indicating no hemispheric lateralization of F0 contour and F0 level processing in this group. The non-lateralized F0 processing may be attributed to the protracted development of tonal processing before school age, reflecting an immature central auditory processing of F0 information in these children compared to adults (Ponton et al., 2002; Moore and Linthicum, 2007; Lazard et al., 2012). Also, the p-MMRs observed in children and MMNs observed in adults may support the hypothesis that p-MMR is a pre-mature form of MMN (Trainor et al., 2001; He et al., 2007, 2009a,b; Cheng et al., 2013; Liu et al., 2014).

Consistent with findings at the attentive stage in Huang et al. (2022), the relative sensitivity to F0 contour and F0 level change in children with CIs was opposite to that in NH children, as reflected by the peak latencies of p-MMRs to F0 contour and F0 level change in the two groups. Note that children with CIs in this study were prelingually deaf, and their hearing experience mainly came from CIs. Thus, the different pattern of F0 processing between NH children and children with CIs probably originated from their different hearing experience. It is well recognized that the way speech sounds being processed in CI systems was different from that in normally functioned cochlea. In CI systems, sound frequency up to 300 Hz is mainly coded and perceived by temporal information in voiced sounds (Shannon, 1983; Zeng, 2002). Although tonal language-oriented speech processing strategies have been developed in recent years, F0 amplitude modulation depth remains inconsistently coded, which results in inconsistent F0 level perception even in the same talkers. However, perception of F0 contour seems not to be affected by the inaccurate F0 amplitude modulation depth (Moore and Carlyon, 2005; Vandali et al., 2013). In this case, it is not surprising that sensitivity to F0 level change is significantly compromised compared to that to F0 contour change.

The unexpected finding in the current study is that the peak latency of p-MMR to F0 contour change in children with CIs was significantly shorter than that to F0 change in NH children. Note that in some of previous studies, F0 contour processing at the attentive stage in Mandarin-speaking children with CIs was reported to be significantly worse than that in their NH peers. For example, Peng et al. (2017) found that compared to NH peers, Mandarin-speaking children with CIs demonstrated less reliance on F0 contours when performing word recognition tasks. Moreover, although Huang et al. (2022) also reported a higher sensitivity to F0 contour change than to F0 level change in Mandarin-speaking preschool children, the overall sensitivity to F0 contour change in children with CIs was, however, significantly compromised compared to that in NH children. In this study, the salient sensitivity to F0 contour change in children with CIs found at the pre-attentive stage in this study may result from the properties of both speech processing strategies in CIs and significant role of F0 in Mandarin Chinese. Limited by speech processing strategies in CI systems, acoustic information (especially F0 cues) available for children with CIs remains insufficient compared to that for NH children. Contour information plays a primary role in Mandarin speech understanding (Lin, 1988). Given the decisive role of contour information in Mandarin Chinese and the limited access to F0 information through CIs, children with CIs may have developed a special “contour processing strategy” for acoustic information use, being highly sensitive to contour information of the incoming sound signals. These discrepant findings on F0 processing in children with CIs between pre-attentive stage and attentive stage may suggests a dissociation on bottom-up and top-down F0 (at least F0 contour) processing. The detection of F0 contour change at the pre-attentive stage resembled a bottom-up processing, in which perception was built on the current input of sensory information (Gibson and Carmichael, 1966). At this stage, the speedy detection of F0 contour change in children with CIs, as reflected by the short peak latency of p-MMR, was consistent with the speculation of “contour processing strategy.” However, at the attentive stage, F0 processing was dominated by the top-down processing, which interpreted of incoming information based on prior knowledge and experience (Gregory, 1970). Given the well-known limitations on F0 transmission in CI systems, it is not surprising that CI users exhibited compromised F0 processing at attentive stage. In future studies, it would be interesting to find out what prevents the interpretation of early and sensitive F0 contour processing in children with CIs from pre-attentive stage to attentive stage.

There are several limitations in the current study. Firstly, the age range of children (3.42–5.7 years) recruited in this work was of a relatively large span, within which language acquisition developed at a high speed (e.g., McCarthy, 1943; Lee et al., 2012). Unfortunately, the sample size in this study was too small to separate into subgroups and investigate the developmental trajectory of F0 processing in children with CIs. Secondly, given the very young age of children, the “attentive” F0 contour and level processing tasks were not performed in the current study, which limited the comparison for current findings with the previous ones. Thirdly, lots of previous studies have demonstrated that acoustic hearing experience or low frequency residual hearing level significantly correlated with pitch perception performance (Tao et al., 2015, 2018, 2022; Liu et al., 2019). However, the detailed aided and unaided hearing thresholds were not well documented across all children in this study, making it difficult to investigate the relationship between hearing level and neural responses to F0 change.

In conclusion, the current study reported for the first time F0 processing at the pre-attentive stage in Mandarin-speaking children with CIs. Investigations on the peak latencies of p-MMRs to F0 contour and F0 level change reveled a higher sensitivity to F0 level change than to F0 contour change in NH children; however, children with CIs were more sensitive to F0 contour change than to F0 level change. The opposite pattern of sensitivities to F0 contour and F0 level change in children with CIs and NH children may indicate different neural mechanisms of F0 processing at pre-attentive stage between these two groups. Moreover, as reflected by the shorter peak latency of p-MMR to F0 contour change in the CI group, detection of F0 contour change in children with CIs was even faster than that of F0 level change in NH children, which contradicted the compromised F0 contour perception at the attentive stage that was reported by some studies (e.g., Xu and Zhou, 2011; Peng et al., 2017; Huang et al., 2022). It would be interesting to find out the representation of F0 contour information in the intermediate process between the pre-attentive and attentive stage in future studies.

## Data availability statement

The raw data supporting the conclusions of this article will be made available by the authors, without undue reservation.

## Ethics statement

The studies involving human participants were reviewed and approved by the Human Research Ethics Committee of the University of Hong Kong. Written informed consent to participate in this study was provided by the participants’ legal guardian/next of kin.

## Author contributions

WH designed and performed experiments, analyzed data, and wrote the manuscript. FC provided the funding for running the study. LW and FC commented on the manuscripts and provided critical revision. All authors discussed the results and implications.

## References

[B1] Ahmmed A. U. Clarke E. M. Adams C. (2008). Mismatch negativity and frequency representational width in children with specific language impairment. *Dev. Med. Child Neurol.* 50 938–944. 10.1111/j.1469-8749.2008.03093.x 18808425

[B2] Benasich A. A. Choudhury N. Friedman J. T. Realpe-Bonilla T. Chojnowska C. Gou Z. (2006). The infant as a prelinguistic model for language learning impairments: Predicting from event-related potentials to behavior. *Neuropsychologia* 44 396–411. 10.1016/j.neuropsychologia.2005.06.004 16054661 PMC1569769

[B3] Berk S. B. (2003). *Sensitive period effects on the acquisition of language: A study of language development.* Storrs, CT: University of Connecticut.

[B4] Boersma P. Weenink D. (2008). *Praat, a system for doing phonetics by computer (version 5.0. 34) [computer software].*

[B5] Chen Y. Wong L. L. Chen F. Xi X. (2014). Tone and sentence perception in young Mandarin-speaking children with cochlear implants. *Int. J. Pediatr. Otorhinolaryngol.* 78 1923–1930. 10.1016/j.ijporl.2014.08.025 25213422

[B6] Cheng Y.-Y. Wu H.-C. Tzeng Y.-L. Yang M.-T. Zhao L.-L. Lee C.-Y. (2013). The development of mismatch responses to Mandarin lexical tones in early infancy. *Dev. Neuropsychol.* 38 281–300. 10.1080/87565641.2013.799672 23862633

[B7] Cheng Y.-Y. Wu H.-C. Tzeng Y.-L. Yang M.-T. Zhao L.-L. Lee C.-Y. (2015). Feature-specific transition from positive mismatch response to mismatch negativity in early infancy: Mismatch responses to vowels and initial consonants. *Int. J. Psychophysiol.* 96 84–94. 10.1016/j.ijpsycho.2015.03.007 25819712

[B8] Cheour M. Shestakova A. Alku P. Ceponiene R. Näätänen R. (2002). Mismatch negativity shows that 3–6-year-old children can learn to discriminate non-native speech sounds within two months. *Neurosci. Lett.* 325 187–190. 10.1016/S0304-3940(02)00269-0 12044652

[B9] Clark J. Yallop C. (1990). *An introduction to phonetics and phonology.* Oxford: Blackwell.

[B10] Conti-Ramsden G. Durkin K. (2012). Language development and assessment in the preschool period. *Neuropsychol. Rev.* 22 384–401. 10.1007/s11065-012-9208-z 22707315

[B11] Debener S. Hine J. Bleeck S. Eyles J. (2008). Source localization of auditory evoked potentials after cochlear implantation. *Psychophysiology* 45 20–24. 10.1111/j.1469-8986.2007.00610.x 17910729

[B12] Dinces E. Chobot-Rhodd J. Sussman E. (2009). Behavioral and electrophysiological measures of auditory change detection in children following late cochlear implantation: A preliminary study. *Int. J. Pediatr. Otorhinolaryngol.* 73 843–851. 10.1016/j.ijporl.2009.03.002 19380166 PMC2688904

[B13] Du X. Sun X. Huang Z. (2005). Introduction of “Study of Chinese language education in CI postoperative rehabilitation”. *Chin. Sci. J. Hear. Speech Rehabil.* 6:12.

[B14] Engström E. Kallioinen P. von Mentzer C. N. Lindgren M. Sahlén B. Lyxell B. (2021). Auditory event-related potentials and mismatch negativity in children with hearing loss using hearing aids or cochlear implants–A three-year follow-up study. *Int. J. Pediatr. Otorhinolaryngol.* 140:110519. 10.1016/j.ijporl.2020.110519 33268013

[B15] Fu M. Wang L. Zhang M. Yang Y. Sun X. (2016). A mismatch negativity study in Mandarin-speaking children with sensorineural hearing loss. *Int. J. Pediatr. Otorhinolaryngol.* 91 128–140. 10.1016/j.ijporl.2016.10.020 27863627

[B16] Gandour J. (1983). Tone perception in Far Eastern languages. *J. Phonetics* 11 149–175. 10.1016/S0095-4470(19)30813-7

[B17] Gibson J. J. Carmichael L. (1966). *The senses considered as perceptual systems*, Vol. 2. Boston, MA: Houghton Mifflin, 44–73.

[B18] Gregory R. L. (1970). *The intelligent eye.* London: Weidenfeld & Nicolson.

[B19] Han D. Liu B. Zhou N. Chen X. Kong Y. Liu H. (2009). Lexical tone perception with HiResolution and HiResolution 120 sound-processing strategies in pediatric Mandarin-speaking cochlear implant users. *Ear Hear.* 30:169. 10.1097/AUD.0b013e31819342cf 19194297 PMC2783178

[B20] He C. Hotson L. Trainor L. J. (2007). Mismatch responses to pitch changes in early infancy. *J. Cogn. Neurosci.* 19 878–892. 10.1162/jocn.2007.19.5.878 17488211

[B21] He C. Hotson L. Trainor L. J. (2009a). Development of infant mismatch responses to auditory pattern changes between 2 and 4 months old. *Eur. J. Neurosci.* 29 861–867. 10.1111/j.1460-9568.2009.06625.x 19200074

[B22] He C. Hotson L. Trainor L. J. (2009b). Maturation of cortical mismatch responses to occasional pitch change in early infancy: Effects of presentation rate and magnitude of change. *Neuropsychologia* 47 218–229. 10.1016/j.neuropsychologia.2008.07.019 18722392

[B23] Henkin Y. Kileny P. R. Hildesheimer M. Kishon-Rabin L. (2008). Phonetic processing in children with cochlear implants: An auditory event-related potentials study. *Ear Hear.* 29 239–249. 10.1097/AUD.0b013e3181645304 18595188

[B24] Hiskey M. S. (1956). A study of the intelligence of deaf and hearing children. *Am. Annal. Deaf* 101 329–339.

[B25] Hochmair I. Nopp P. Jolly C. Schmidt M. Schößer H. Garnham C. (2006). MED-EL cochlear implants: State of the art and a glimpse into the future. *Trends Amplif.* 10 201–219. 10.1177/1084713806296720 17172548 PMC4111377

[B26] Huang W. Wong L. L. Chen F. (2022). Just-noticeable differences of fundamental frequency change in mandarin-speaking children with cochlear implants. *Brain Sci.* 12:443. 10.3390/brainsci12040443 35447975 PMC9031813

[B27] Huang W. Wong L. L. Chen F. Liu H. Liang W. (2020). Effects of fundamental frequency contours on sentence recognition in Mandarin-speaking children with cochlear implants. *J. Speech Lang. Hear. Res.* 63 3855–3864. 10.1044/2020_JSLHR-20-00033 33022190

[B28] Huang W.-T. Nan Y. Dong Q. Liu C. (2015). Just-noticeable difference of tone pitch contour change for Mandarin congenital amusics. *J. Acoust. Soc. Am.* 138 EL99–EL104. 10.1121/1.4923268 26233070

[B29] Ibraheem O. A. Kolkaila E. A. Nada E. H. Gad N. H. (2020). Auditory cortical processing in cochlear-implanted children with different language outcomes. *Eur. Arch. Otorhinolaryngol.* 277 1875–1883. 10.1007/s00405-020-05958-0 32270327

[B30] Kileny P. R. Boerst A. Zwolan T. (1997). Cognitive evoked potentials to speech and tonal stimuli in children with implants. *Otolaryngol. Head Neck Surg.* 117 161–169. 10.1016/S0194-5998(97)70169-4 9334760

[B31] Lazard D. S. Collette J. L. Perrot X. (2012). Speech processing: From peripheral to hemispheric asymmetry of the auditory system. *Laryngoscope* 122 167–173. 10.1002/lary.22370 22095864

[B32] Lee C.-Y. Yen H.-l Yeh P.-w Lin W.-H. Cheng Y.-Y. Tzeng Y.-L. (2012). Mismatch responses to lexical tone, initial consonant, and vowel in Mandarin-speaking preschoolers. *Neuropsychologia* 50 3228–3239. 10.1016/j.neuropsychologia.2012.08.025 22981563

[B33] Liang M. Zhang X. Chen T. Zheng Y. Zhao F. Yang H. (2014). Evaluation of auditory cortical development in the early stages of post cochlear implantation using mismatch negativity measurement. *Otol. Neurotol.* 35 e7–e14. 10.1097/MAO.0000000000000181 24335940

[B34] Light G. A. Swerdlow N. R. Braff D. L. (2007). Preattentive sensory processing as indexed by the MMN and P3a brain responses is associated with cognitive and psychosocial functioning in healthy adults. *J. Cogn. Neurosci.* 19 1624–1632. 10.1162/jocn.2007.19.10.1624 18271737 PMC2562660

[B35] Lin M. (1988). The acoustic characteristics and perceptual cues of tones in Standard Chinese. *Chin. Yuwen* 204 182–193.

[B36] Liu H.-M. Chen Y. Tsao F.-M. (2014). Developmental changes in mismatch responses to Mandarin consonants and lexical tones from early to middle childhood. *PLoS One* 9:e95587. 10.1371/journal.pone.0095587 24755999 PMC3995781

[B37] Liu Y. L. (1989). *Research on the Chinese proficiency test.* Beijing: Modern Press Co., Ltd.

[B38] Liu Y. W. Tao D. D. Chen B. Cheng X. Shu Y. Galvin J. J. III (2019). Factors affecting bimodal benefit in pediatric Mandarin-speaking Chinese cochlear implant users. *Ear Hear.* 40:1316. 10.1097/AUD.0000000000000712 30882534 PMC6745007

[B39] Luck S. J. (2014). *An introduction to the event-related potential technique.* Cambridge: MIT press.

[B40] Maurer U. Bucher K. Brem S. Brandeis D. (2003). Development of the automatic mismatch response: From frontal positivity in kindergarten children to the mismatch negativity. *Clin. Neurophysiol.* 114 808–817. 10.1016/S1388-2457(03)00032-4 12738427

[B41] McCarthy D. (1943). “Child behavior and development: A course of representative studies,” In *Language development in the preschool child*, eds Barker R. G. Kounin J. S. Wright H. F. (New York, NY: McGraw-Hill), 107–128. 10.1037/10786-007

[B42] Moore B. C. Carlyon R. P. (2005). “Perception of pitch by people with cochlear hearing loss and by cochlear implant users,” in *Pitch*, eds Plack C. J. Fay R. R. Oxenham A. J. Popper A. N. (New York, NY: Springer), 234–277. 10.1007/0-387-28958-5_7

[B43] Moore J. K. Linthicum F. H. Jr. (2007). The human auditory system: A timeline of development. *Int. J. Audiol.* 46 460–478. 10.1080/14992020701383019 17828663

[B44] Morr M. L. Shafer V. L. Kreuzer J. A. Kurtzberg D. (2002). Maturation of mismatch negativity in typically developing infants and preschool children. *Ear Hear.* 23 118–136. 10.1097/00003446-200204000-00005 11951848

[B45] Näätänen R. (2001). The perception of speech sounds by the human brain as reflected by the mismatch negativity (MMN) and its magnetic equivalent (MMNm). *Psychophysiology* 38 1–21. 10.1111/1469-8986.3810001 11321610

[B46] Näätänen R. (2008). Mismatch negativity (MMN) as an index of central auditory system plasticity. *Int. J. Audiol.* 47(Suppl. 2) S16–S20. 10.1080/14992020802340116 19012108

[B47] Näätänen R. Gaillard A. W. Mäntysalo S. (1978). Early selective-attention effect on evoked potential reinterpreted. *Acta Psychol.* 42 313–329. 10.1016/0001-6918(78)90006-9 685709

[B48] Näätänen R. Paavilainen P. Rinne T. Alho K. (2007). The mismatch negativity (MMN) in basic research of central auditory processing: A review. *Clin. Neurophysiol.* 118 2544–2590. 10.1016/j.clinph.2007.04.026 17931964

[B49] Nan Y. Huang W. T. Wang W.-J. Liu C. Dong Q. (2016). Subgroup differences in the lexical tone mismatch negativity (MMN) among Mandarin speakers with congenital amusia. *Biol. Psychol.* 113 59–67. 10.1016/j.biopsycho.2015.11.010 26638759

[B50] Nin J.-Z. (2021). *Key data from the seventh national census.* Available online at: http://www.stats.gov.cn/tjsj/zxfb/202105/t20210510_1817176.html (accessed October 24, 2022).

[B51] Pang E. Edmonds G. Desjardins R. Khan S. Trainor L. Taylor M. (1998). Mismatch negativity to speech stimuli in 8-month-old infants and adults. *Int. J. Psychophysiol.* 29 227–236. 10.1016/S0167-8760(98)00018-X 9664230

[B52] Partanen E. Torppa R. Pykäläinen J. Kujala T. Huotilainen M. (2013). Children’s brain responses to sound changes in pseudo words in a multifeature paradigm. *Clin. Neurophysiol.* 124 1132–1138. 10.1016/j.clinph.2012.12.005 23317916

[B53] Peng S.-C. Lu H.-P. Lu N. Lin Y.-S. Deroche M. L. Chatterjee M. (2017). Processing of acoustic cues in lexical-tone identification by pediatric cochlear-implant recipients. *J. Speech Lang. Hear. Res.* 60 1223–1235. 10.1044/2016_JSLHR-S-16-0048 28388709 PMC5755546

[B54] Perez V. B. Tarasenko M. Miyakoshi M. Pianka S. T. Makeig S. D. Braff D. L. (2017). Mismatch negativity is a sensitive and predictive biomarker of perceptual learning during auditory cognitive training in schizophrenia. *Neuropsychopharmacology* 42 2206–2213. 10.1038/npp.2017.25 28139679 PMC5603809

[B55] Ponton C. Eggermont J. J. Khosla D. Kwong B. Don M. (2002). Maturation of human central auditory system activity: Separating auditory evoked potentials by dipole source modeling. *Clin. Neurophysiol.* 113 407–420. 10.1016/S1388-2457(01)00733-7 11897541

[B56] Qi B. Liu Z. Gu X. Liu B. (2017). Speech recognition outcomes in Mandarin-speaking cochlear implant users with fine structure processing. *Acta Oto Laryngol.* 137 286–292. 10.1080/00016489.2016.1230680 27701966

[B57] Schwade L. F. Didoné D. D. Sleifer P. (2017). Auditory evoked potential mismatch negativity in normal-hearing adults. *Int. Arch. Otorhinolaryngol.* 21 232–238. 10.1055/s-0036-1586734 28680490 PMC5495584

[B58] Segalowitz S. J. Barnes K. L. (1993). The reliability of ERP components in the auditory oddball paradigm. *Psychophysiology* 30 451–459. 10.1111/j.1469-8986.1993.tb02068.x 8416071

[B59] Shafer V. L. Morr M. L. Kreuzer J. A. Kurtzberg D. (2000). Maturation of mismatch negativity in school-age children. *Ear Hear.* 21 242–251. 10.1097/00003446-200006000-00008 10890733

[B60] Shannon R. V. (1983). Multichannel electrical stimulation of the auditory nerve in man. I. Basic psychophysics. *Hear. Res.* 11 157–189. 10.1016/0378-5955(83)90077-1 6619003

[B61] Sharma A. Campbell J. Cardon G. (2015). Developmental and cross-modal plasticity in deafness: Evidence from the P1 and N1 event related potentials in cochlear implanted children. *Int. J. Psychophysiol.* 95 135–144. 10.1016/j.ijpsycho.2014.04.007 24780192 PMC4209331

[B62] Singh S. Liasis A. Rajput K. Towell A. Luxon L. (2004). Event-related potentials in pediatric cochlear implant patients. *Ear Hear.* 25 598–610. 10.1097/00003446-200412000-00008 15604920

[B63] Tao D. D. Liu J. S. Yang Z. D. Wilson B. S. Zhou N. (2018). Bilaterally combined electric and acoustic hearing in Mandarin-speaking listeners: The population with poor residual hearing. *Trends Hear.* 22:2331216518757892. 10.1177/2331216518757892 29451107 PMC5818091

[B64] Tao D. D. Zhang Y. M. Liu H. Zhang W. Xu M. Galvin J. J. III (2022). The P300 auditory event-related potential may predict segregation of competing speech by bimodal cochlear implant listeners. *Front. Neurosci.* 16:888596. 10.3389/fnins.2022.888596 35757527 PMC9226716

[B65] Tao D. Deng R. Jiang Y. Galvin J. J. III Fu Q. J. Chen B. (2015). Melodic pitch perception and lexical tone perception in Mandarin-speaking cochlear implant users. *Ear Hear.* 36:102. 10.1097/AUD.0000000000000086 25099401 PMC4272610

[B66] Tiitinen H. May P. Reinikainen K. Näätänen R. (1994). Attentive novelty detection in humans is governed by pre-attentive sensory memory. *Nature* 372 90–92. 10.1038/372090a0 7969425

[B67] Tong X. McBride C. Zhang J. Chung K. K. Lee C.-Y. Shuai L. (2014). Neural correlates of acoustic cues of English lexical stress in Cantonese-speaking children. *Brain Lang.* 138 61–70. 10.1016/j.bandl.2014.09.004 25310025

[B68] Torppa R. Salo E. Makkonen T. Loimo H. Pykäläinen J. Lipsanen J. (2012). Cortical processing of musical sounds in children with cochlear implants. *Clin. Neurophysiol.* 123 1966–1979. 10.1016/j.clinph.2012.03.008 22554786

[B69] Trainor L. J. Samuel S. S. Desjardins R. N. Sonnadara R. R. (2001). Measuring temporal resolution in infants using mismatch negativity. *Neuroreport* 12 2443–2448. 10.1097/00001756-200108080-00031 11496126

[B70] Uhlén I. Engström E. Kallioinen P. Nakeva von Mentzer C. Lyxell B. Sahlén B. (2017). Using a multi-feature paradigm to measure mismatch responses to minimal sound contrasts in children with cochlear implants and hearing aids. *Scand. J. Psychol.* 58 409–421. 10.1111/sjop.12391 28901574

[B71] Uhler K. Hunter S. Gilley P. M. (2021). Mismatched response predicts behavioral speech discrimination outcomes in infants with hearing loss and normal hearing. *Infancy* 26 327–348. 10.1111/infa.12386 33481354 PMC7935319

[B72] Vandali A. Sly D. Cowan R. Van Hoesel R. (2013). Pitch and loudness matching of unmodulated and modulated stimuli in cochlear implantees. *Hear. Res.* 302 32–49. 10.1016/j.heares.2013.05.004 23685148

[B73] Wang X.-D. Wang M. Chen L. (2013). Hemispheric lateralization for early auditory processing of lexical tones: Dependence on pitch level and pitch contour. *Neuropsychologia* 51 2238–2244. 10.1016/j.neuropsychologia.2013.07.015 23911775

[B74] Wei C. Cao K. Jin X. Chen X. Zeng F.-G. (2007). Psychophysical performance and Mandarin tone recognition in noise by cochlear implant users. *Ear Hear.* 28(Suppl. 2) 62S. 10.1097/AUD.0b013e318031512c 17496650 PMC2674760

[B75] Wilson B. S. (2006). Speech processing strategies. *Cochlear implants* 2 21–69.

[B76] Xu L. Zhou N. (2011). “Tonal languages and cochlear implants,” in *Auditory prostheses*, eds Zeng F. G. Popper A. N. Fay R. R. (New York, NY: Springer), 341–364. 10.1007/978-1-4419-9434-9_14

[B77] Yip M. (2002). *Tone.* Cambridge: Cambridge University Press. 10.1017/CBO9781139164559

[B78] Zeng F.-G. (2002). Temporal pitch in electric hearing. *Hear. Res.* 174 101–106. 10.1016/S0378-5955(02)00644-5 12433401

[B79] Zhang L. Wang J. Hong T. Li Y. Zhang Y. Shu H. (2018). Mandarin-speaking, kindergarten-aged children with cochlear implants benefit from natural f 0 patterns in the use of semantic context during speech recognition. *J. Speech Lang. Hear. Res.* 61 2146–2152. 10.1044/2018_JSLHR-H-17-0327 30073305

